# Determination of Bioactive Components in Chrysanthemum Tea (Gongju) Using Hyperspectral Imaging Technique and Chemometrics

**DOI:** 10.3390/foods13244145

**Published:** 2024-12-21

**Authors:** Yunpeng Wei, Huiqiang Hu, Minghua Yuan, Huaxing Xu, Xiaobo Mao, Yuping Zhao, Luqi Huang

**Affiliations:** 1 School of Electrical and Information Engineering, Zhengzhou University, Zhengzhou 450001, China; weiyunpeng@gs.zzu.edu.cn (Y.W.); huhuiqiang_zzu@163.com (H.H.); xuhuaxing@zzu.edu.cn (H.X.); huangluqi01@126.com (L.H.); 2 Research Center for Intelligent Science and Engineering Technology of Traditional Chinese Medicine, Zhengzhou 450001, China; 3 Department of Pharmacy, Zhengzhou Shuqing Medical College, Zhengzhou 450064, China; yuanmh123@yeah.net; 4 China Academy of Chinese Medical Sciences, Beijing 100700, China

**Keywords:** chrysanthemum tea, hyperspectral imaging, wavelength selection, particle swarm optimization, ensemble learning

## Abstract

The bioactive components of chrysanthemum tea are an essential indicator in evaluating its nutritive and commercial values. Combining hyperspectral imaging (HSI) with key wavelength selection and pattern recognition methods, this study developed a novel approach to estimating the content of bioactive components in chrysanthemums, including the total flavonoids (TFs) and chlorogenic acids (TCAs). To determine the informative wavelengths of hyperspectral images, we introduced a variable similarity regularization term into particle swarm optimization (denoted as VSPSO), which can focus on improving the combinatorial performance of key wavelengths and filtering out the features with higher collinearity simultaneously. Moreover, considering the underlying relevance of the phytochemical content and the exterior morphology characteristics, the spatial image features were also extracted. Finally, an ensemble learning model, LightGBM, was established to estimate the TF and TCA contents using the fused features. Experimental results indicated that the proposed VSPSO achieved a superior accuracy, with R^2^ scores of 0.9280 and 0.8882 for TF and TCA prediction. Furthermore, after the involvement of spatial image information, the fused spectral–spatial features achieved the optimal model accuracy on LightGBM. The R^2^ scores reached 0.9541 and 0.9137, increasing by 0.0308–0.1404 and 0.0181–0.1066 in comparison with classical wavelength-related methods and models. Overall, our research provides a novel method for estimating the bioactive components in chrysanthemum tea accurately and efficiently. These discoveries revealed the potential effectiveness for constructing feature fusion in HSI-based practical applications, such as nutritive value evaluation and heavy metal pollution detection, which will also facilitate the development of quality detection in the food industry.

## 1. Introduction

Tea, one of the most common beverages and consumer goods, has abundant phytochemicals and minerals, such as polyphenols, theine, calcium, and potassium, which are beneficial to human health [1]. As one of the most representative flower teas, Gongju (*Dendranthema morifolium* (Ramat) Tzvel. cv. Gongju), one of the four famous Chinese chrysanthemums, is mainly produced in Huangshan city in China, with a cultivation history of over seven hundred years [2]. Due to the unique tea aroma, it is well received as a flower tea and has been a daily necessity [3]. Besides the external factors of taste and luster, chrysanthemum tea is rich in internal nutritional value, which is also a concern for consumers. Previous studies have shown that chrysanthemum contains many bioactive components with health benefits, including flavonoids [4], chlorogenic acids [5], polysaccharides [6], and essential oils [7], revealing enormous potential in the fields of health care, the food industry, and pharmaceuticals [8]. The contents of these phytochemicals determine the quality of chrysanthemum tea directly [9]. However, there is no obvious difference in the appearance and smell of different qualities of chrysanthemum tea, thereby leading to adulterated products emerging in the market. For instance, the sulfur fumigation process can be used to enhance the chrysanthemum appearance and counterfeit quality goods, which deteriorates the antioxidant activity and leads to the loss of some bioactive constituents [10]. Consequently, the development of an accurate approach for quality assessment of chrysanthemum tea is of great importance for consumer interest protection and market supervision [11].

With the development of modern science and technology, many physicochemical techniques for measuring the content of bioactive constituents have emerged and have become the mainstream in quantitative analysis research, primarily including chromatography [12] and spectroscopy [13]. The representative chromatography method includes high performance liquid chromatography (HPLC) [14], gas chromatography (GC) [15], and capillary electrophoresis (CE) [16]. The spectroscopy technique includes the ultraviolet spectrum (UVS) [17], nuclear magnetic resonance (NMR) [18], and laser-introduced breakdown spectroscopy (LIBS) [19]. Despite the objective and reliable properties, these methods inevitably suffer from the problems of the destruction of the integrity of samples, being time-consuming, producing residual chemical reagents, and requiring specific experimental equipment [20]. To alleviate this problem, in recent years, some studies have highlighted the feasibility of the near-infrared spectroscopy (NIRS) technique for rapid and effective qualitative and quantitative analysis, such as nitrogen level detection in *Brassica juncea* [21], mineral content measurement in perilla [22], and the estimation of free fatty acid concentration in oil palm fruit [23]. However, due to the characteristic of specific point detection, it may be inadequate for capturing the heterogeneity of entire samples, thereby deteriorating the performance of subsequent chemometric analysis [24,25].

Combining spectroscopy with the imaging technique, hyperspectral imaging (HSI) can collect more abundant spectral information within a broader wavelength range as well as pixel-based spatial image information [26], providing a promising solution for improving the estimation accuracy and carrying out a more comprehensive analysis [27]. Many successful applications related to quality detection and food safety monitoring in the food industry have emerged, such as physical trait and composition detection in maize kernel [28], monitoring interior composition variation in cheese ripening [29], and assessing the total soluble solids and firmness of cherries [30]. Regarding the HSI-based quality assessment for chrysanthemum tea, Long et al. constructed a bagging classification tree-radial basis function network (BAGCT-RBFN) to carry out the geographical origin traceability of chrysanthemums [31]. Combining the traditional support vector machine (SVR), partial least squares regression (PLSR), and convolutional neural network (CNN), He et al. established a quantitative analysis model for five micro-components [32]. However, among these existing applications, there are still several limitations despite the achieved reasonable accuracy, such as insufficient exploitation for key wavelength selection and spatial image feature extraction. Consequently, an effective data processing method for exploiting spectral–spatial features sufficiently is also essential to HSI-related application, especially the development of online lightweight equipment.

Motivated by these factors, this study aims to estimate the bioactive component contents of chrysanthemum tea using hyperspectral imaging and chemometrics. Specifically, (1) to enhance the performance of key wavelength selection as well as the dimensionality reduction, a variable similarity regularization term is introduced to particle swarm optimization. It concentrates not only on selecting informative spectral features but also on removing the redundant ones with higher similarity simultaneously, thereby representing the original hyperspectral information using relatively fewer spectral features. (2) Considering the potential relevance of the phytochemical content and the exterior morphology characteristics, we also extract multiple pieces of spatial image information, including the gray-level concurrence matrix (GLCM), texture, and color features. (3) To achieve a better prediction accuracy, an ensemble learning-based multivariate regression model (light gradient boosting machine, LightGBM) [33] is built for bioactive component content estimation of chrysanthemum tea according to the obtained fused features.

## 2. Materials and Methods

**Figure 1:**
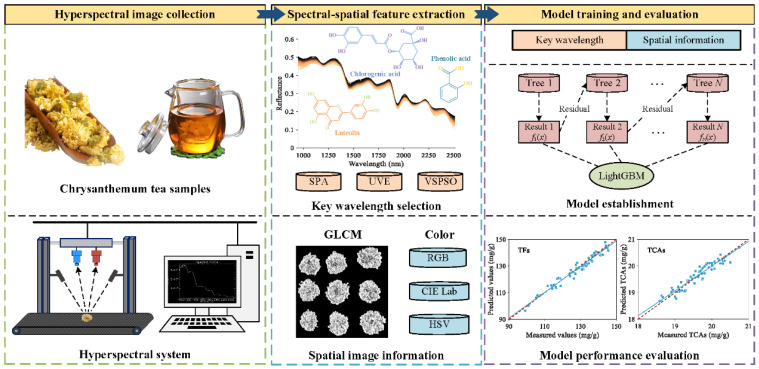
Flowchart of chrysanthemum component prediction by the hyperspectral imaging technique and chemometrics.

Figure 1 depicts the overall procedure of the prediction of bioactive component contents in chrysanthemum tea. Specifically, the main steps are described as follows.

### 2.1. Sample Preparation

In this study, the dried flowers of Huangshan Gongju were used as the experimental samples of chrysanthemum tea, which originated in Shexian County, Huangshan City, Anhui, and were provided by Huirentang Pharmaceutical Co., Ltd, Huangshan, Anhui, China. All chrysanthemum tea samples were meticulously weighed and grouped with 10 g per group. Among them, 140 groups were harvested at the blooming period in October 2022 for calibration and chemometric model training, while another 60 groups collected in 2023 were used to constitute the external test set to verify the prediction performance of constructed models. After grouping, all samples were stored in sealable bags and placed in dry conditions for subsequent hyperspectral image acquisition and physicochemical analysis.

### 2.2. Hyperspectral Image Acquisition and Data Correction

The hyperspectral images were captured by the HySpex spectrometer series (HySpex VNIR-1800/SWIR-384, Norsk Elektro Optikk, Oslo, Norway) (VNIR: visible and near-infrared ray; SWIR: short-wave infrared ray). To guarantee a stable and effective illumination, it is equipped with two 150 W tungsten halogen lamps (H-LAM, Norsk Elektro Optikk, Oslo, Norway). The VNIR lens covers the wavelength range from 410 to 990 nm (108 bands), while the SWIR can capture hyperspectral images with a spectral range within 948–2513 nm (288 bands). In this study, the SWIR region was used to conduct the spectra extraction and key wavelength selection, and the VNIR data covering the visible spectrum information were used to extract the spatial image features of texture and color information.

**Figure 2:**
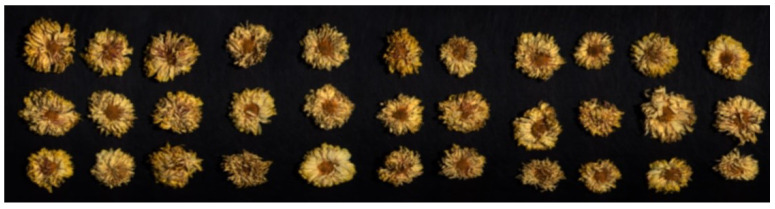
The visual representation of the samples of chrysanthemum tea. The false-color image was formed using visible wavelengths at 442.92, 545.85, and 621.70 nm.

To decrease the stray light noise, the process of hyperspectral image acquisition was carried out in a dark room. The system and two halogen lamps were activated in advance to preheat for 30 min. Regarding camera parameters, the integration time of VNIR and SWIR was 4000 and 4500 μs, and the frame period was 18,000 and 46,928 μs, respectively. When collecting data, all samples within each group were evenly laid onto the conveyor and driven by a computer system with a constant speed of 2.0 mm/s. A visual representation of collected samples is depicted in Figure 2, in which the false-color image was formed using the bands at 442.92, 545.85, and 621.70 nm.

During the capture, the image is susceptible to the interference caused by external noise such as the charge-coupled device’s (CCD’s) camera dark current. To enhance the data purity, the data correction for raw hyperspectral images was essential, and is formulated as follows:

(1) $$I_{new}=\frac{I_{raw}-I_{dark}}{I_{white}-I_{dark}}$$

where *I_raw_* and *I_new_* denote the raw and corrected hyperspectral images. A Teflon whiteboard was employed to collect the white reference image *I_white_*, while the dark reference image (*I_dark_*) was acquired by closing the lens cover.

### 2.3. Determination of Reference Values of Total Flavonoids and Chlorogenic Acid Content

#### 2.3.1. Equipment and Chemicals

Materials included an ultraviolet–visible (UV) spectrophotometer of UV-752PC and electronic balance (Shanghai Precision Scientific Instrument Co., Ltd., Shanghai, China), an HPLC Agilent Technologies 1260 Infinity system (Shanghai Agilent Technology Co., Ltd., Shanghai, China), and an ultrasonic cleaner (Kunshan Ultrasonic Instrument Co., Ltd., Suzhou, Jiangsu, China), Methanol, sodium nitrite, aluminum nitrate, sodium hydroxide, acetonitrile, phosphoric acid aqueous, standard reference materials of rutin (purity ≥ 95%), and chlorogenic acid were all provided by Aladdin Bio-Chem Technology Co., Ltd., Shanghai, China.

#### 2.3.2. Determination of Total Flavonoid Content

UV spectrophotometry was employed to quantify the total flavonoids (TFs) of chrysanthemum tea samples. First, 50 mg of standard rutin were weighed accurately and dissolved in 50% methanol to obtain the standard solution with a concentration of 0.2 mg/mL. Then, specific volumes (1, 2, 3, 4, 5, and 6 mL) of the standard solution were measured meticulously and added to a 25 mL volumetric flask, and an appropriate amount of 50% methanol was added. They were mixed with 1 mL of 5% sodium nitrite solution, 1 mL of 10% aluminum nitrate solution, and 10 mL of 4% sodium hydroxide solution, respectively; shaken well; and left to stand for 6 min each time. Additional 50% methanol was added to adjust the volume to 25 mL. With the 50% methanol as blank control, the absorbance at the wavelength of 510 nm was measured to establish the standard curve. For the test solution preparation, 1 g of chrysanthemum powder was weighed, dissolved in 100 mL of 50% methanol, heated, and refluxed for extraction for 1 h. Next, 10 mL of this solution were measured accurately and diluted with 100 mL 50% methanol as the test solution. Finally, the concentration of test solution as well as the flavonoid content could be determined by measuring and comparing the absorbance at a wavelength of 510 nm with the established standard curve.

#### 2.3.3. Determination of Total Chlorogenic Acid Content

The reference value of total chlorogenic acid (TCA) content was measured by the HPLC technique. For standard solution preparation, an appropriate amount of standard reference material of chlorogenic acid was weighed accurately and dissolved in 50% methanol to obtain the standard solution, with 50 μg/mL. Next, 0.5 g of the chrysanthemum powder was accurately weighed into an Erlenmeyer flask, and 25 mL of 50% methanol were added to the Erlenmeyer flask. After 40 min of the ultrasonic process (300 W, 45 kHz), the solution was weighed again and the lost weight was compensated for with 50% methanol, shaken well, and filtered to obtain filtrate as test solution. Finally, chromatography analysis was performed on the HPLC Agilent Technologies 1260 Infinity system. A C18 column (4.6 mm × 250 mm) thermostated at 25 °C was used to separate the chlorogenic acid components. The mobile phase of A and B was a mixture of acetonitrile and 0.4% phosphoric acid aqueous solution with a ratio of 2:8. The detection wavelength was 327 nm and the flow rate was 0.8 mL/min, with an injection volume of 10 μL.

### 2.4. Spectra Extraction and Preprocessing

Spectra extraction denotes the operation to extract spectral data from the corrected images [34]. During this process, the background separation is employed to remove the background interference, and the whole sample surface is defined as the region of interest (ROI). The spectra of all pixels within the ROI are averaged as spectral data of the corresponding sample. Moreover, to decrease the interference of irrelevant noise, the spectral preprocessing operation is essential. A joint data preprocessing of Savitzky–Golay convolution smoothing (SGCS) and multiplicative scatter correction (MSC) was used in this study. By fitting the spectrum curves using polynomial regression, the spectral smoothing characteristic could be improved by SGCS [35]. Additionally, the multiplicative scatter impacts caused by uneven surface could be decreased by constructing a linear regression transformation between spectral data and averaged spectra in MSC [36].

### 2.5. Feature Extraction Process

Feature extraction aims to obtain valuable information contributing to improving the model prediction performance. With its informative features, it can represent the object information better using a small amount of data and reveal their underlying correlation. To exploit the useful information sufficiently, both the key wavelength and the spatial image information are extracted from the hyperspectral data in this study.

#### 2.5.1. Optimal Wavelength Selection via VSPSO

Particle swarm optimization (PSO) is a population-based global optimization technique, and is employed to search for the most appropriate key wavelengths [37]. First, a random population is generated by the binary encoding strategy, in which “1” and “0” denote whether the corresponding band is selected or not. That is, each encoding particle represents one candidate of the band subset. Next, these particles are updated iteratively using the following equations:

(2) $$\begin{array}{c} V_{kd}^{t+1}=\omega V_{kd}^{t}+c_{1}r_{1}\left({pbest}_{kd}^{t}-X_{kd}^{t}\right)+c_{2}r_{2}\left({gbest}_{d}^{t}-X_{kd}^{t}\right)\\ X_{kd}^{t+1}=X_{kd}^{t}+V_{kd}^{t+1} \end{array}$$

where $X_{kd}^{t}$ and $V_{kd}^{t}$ are the position and corresponding velocity of the *k*th particle at the *d*th dimension in the *t*th iteration, respectively; *ω* ∈ (0, 1) denotes the inertia weight; *c*_1_ and *c*_2_ are the learning factors; *r*_1_ and *r*_2_ are random coefficients of (0, 1); and *pbest_k_* and *gbest* are the personal best position and global best position found by the *k*th particle and the whole population.

The fitness function is used to evaluate the particles during the iterations. In this study, a novel fitness function with a regularization term based on variable similarity was constructed (denoted as VSPSO), which is formulated as the follows:

(3) $$\mathrm{Min}\;f\left(X_{k}\right)=\sqrt{\frac{\sum_{i=1}^{n}{\left({\hat{y}}_{i}-y_{i}\right)}^{2}}{n}+\lambda \frac{l}{L\sum_{a,b}{e^{\left\|w_{a}-w_{b}\right\|}}^{2}}}$$

where the first item is the root mean square error (RMSE), representing the prediction error using the selected wavelength subset *X_k_*. The second item is the regularization term, in which l is the number of wavelengths contained in *X_k_*, and ${e^{\left\|w_{a}-w_{b}\right\|}}^{2}$ (*w_a_*, *w_b_* ∈ *X_k_*, *a* ≠ *b*) denotes the feature similarity of wavelength *w_a_* and *w_b_* based on Euclidean distance. Hence, the minimization of the regularization term can alleviate the feature redundancy and reduce the variable dimension. 0 < *λ* < 1 is the coefficient to balance both items. That is, the proposed VSPSO takes advantage of the combinatorial optimization of population-based methods while taking into account the reduction of prediction error and feature dimension.

#### 2.5.2. Spatial Image Feature Extraction

The external morphological characteristics also contain useful information contributing to the quantitative analysis [38]. To explore the potential relevance between them, the spatial image information of a hyperspectral image is extracted, including the color features and texture information based on the gray-level concurrence matrix (GLCM).

The color features are extracted from 3-color space, including RGB (R: red; G: green; B: blue), CIE L*a*b* (L*: lightness; a*: chroma of red–green; b*: chroma of yellow–blue), and HSV (H: hue; S: saturation; V: value) [39]. When extracting the 9 channels of color information, all pixels within the image are averaged to alleviate the interference caused by uneven color fluctuation [40].

The GLCM can represent the textural feature by describing the correlation and spatial distribution of grayscale pixels [41]. Five types of GLCM-based texture characteristics of angular second moment (ASM), contrast, dissimilarity, energy, and homogeneity were extracted in this study. For each of them, the eigenvalues of four angles (0°, 45°, 90°, 135°) were averaged for rotation invariance. Specifically, the step length and assigned gray level were set to 1 and 64, respectively.

### 2.6. Model Building and Evaluation

#### 2.6.1. Conventional Models

Support vector regression (SVR) is a supervised multivariate regression model [42]. Its base principle is to build an optimal hyperplane for data fitting in high-dimensional feature space. During this process, the tolerance of error and the hyperplane shape are adjusted by two key parameters of penalty (*C*) and kernel efficient (*γ*), respectively [43]. Specifically, both parameters are determined by a grid search based on 5-fold cross-validation with the parameter grid of *C* ∈ [0, 10] and *γ* ∈ [5, 5000]. Partial least squares regression (PLSR) is also a nonlinearity regression model, which integrates the principal component and multivariate regression analysis. It can alleviate the feature collinearity problem and extract the latent variables (Lvs) by considering the mutual correlation of feature and dependent variable matrices [44]. Similarly, the most appropriate LVs are determined in the range [1, 60]. One-dimensional convolutional neural network (1DCNN) is a prevailing deep learning-based model for dealing with spectral data [45]. As a variant of conventional CNNs, it can extract the local patterns and explore the inherent dependency of sequential features, thereby presenting outstanding performance in many complex tasks. In this study, the built 1DCNN begins with the input layer, comprising three convolutional layers with ReLU activation function, two max-pooling layers, a fully connected layer, and an output layer.

#### 2.6.2. LightGBM

Light gradient boosting machine (LightGBM) belongs to the category of gradient boosting decision tree (GBDT), comprising three essential strategies of gradient-based one-side sampling (GOSS), exclusive feature bundling (EFB), and leaf-wise growth strategy [46]. Assume an *m*-dimensional dataset with *n* samples as ***S****^n^*^×*m*^, GOSS first calculates the sample gradient and ranks the samples in descending order. The top *a* × 100% samples are merged to subset *A*, while the remaining (1 − *a*) × 100% instances with a smaller gradient constitute subset *A^c^*. Then, a new sample subset *B* is composed of the *b* × |*A^c^*| instances sampled from *A^c^*. Finally, according to *A* ∪ *B*, a variance gain of feature *j* at point *d*, ${\tilde{V}}_{j}\left(d\right)$ is calculated with the following equation to split the instances:

(4) $${\tilde{V}}_{j}\left(d\right)=\frac{1}{n}\left(\frac{({\sum_{x_{i}\in A_{l}}g_{i}+\frac{1-a}{b}\sum_{x_{i}\in B_{l}}g_{i})}^{2}}{n_{l}^{j}(d)}+\frac{({\sum_{x_{i}\in A_{r}}g_{i}+\frac{1-a}{b}\sum_{x_{i}\in B_{r}}g_{i})}^{2}}{n_{r}^{j}(d)}\right)$$

where *g* is the sample gradient, *A_l_* = {*x_i_* ∈ *A*|*x_ij_* ≤ *d*}, *A_r_* = {*x_i_* ∈ *A*|*x_ij_* > *d*} (*B_l_* and *B_r_* are similar to them), and (1 − *a*)/*b* is the gradient normalization coefficient. Hence, the splitting nodes are determined using parts rather than the total number of instances.

**Figure 3:**
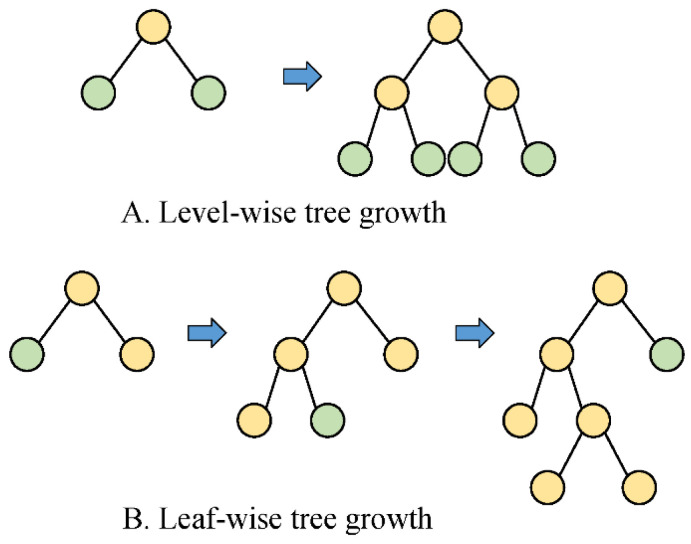
The level-wise and leaf-wise tree growth strategies. (**A**) Level- wise tree growth. (**B**) Leaf-wise tree growth.

EFB defines the exclusive feature when dealing with the high-dimensional problems and allows them to be bundled into a merged feature. In conjunction with GOSS, the model efficiency can be enhanced markedly by simplifying the data structure from the aspects of sample size and dimension. Moreover, instead of the level-wise way, leaf-wise growth is employed to avoid the unessential splitting of leaf nodes, thereby alleviating overfitting. Figure 3 depicts the difference in the level-wise and leaf-wise growth strategies. More detailed information on LightGBM can be found in [33].

#### 2.6.3. Evaluation Metrics for Model Performance

Four metrics were employed to evaluate the model performance for estimating the total flavonoids and chlorogenic acid contents, including the determination coefficient (R^2^), root mean square error (RMSE), mean absolute error (MAE), and residual prediction deviation (RPD) [20].

Assume the actual values *y* = [*y*_1_, *y*_2_, ..., *y_n_*] and corresponding predicted values $\hat{y}=[{\hat{y}}_{1},\;{\hat{y}}_{2},\;\ldots ,\;{\hat{y}}_{n}]$, the evaluation metrics are formulated as follows:

$$R^{2}=1-\frac{\sum_{i=1}^{n}({y_{i}-\hat{y}}_{i}{)}^{2}}{\sum_{i=1}^{n}(y_{i}-\bar{y}{)}^{2}}$$

(5) $$RMSE=\sqrt{\frac{1}{n}\sum_{i=1}^{n}({y_{i}-\hat{y}}_{i}{)}^{2}}$$

$$MAE=\frac{1}{n}\sum_{i=1}^{n}\left|{y_{i}-\hat{y}}_{i}\right|$$

$$RPD=\frac{SD}{RMSE}$$

where $\bar{y}$ is the average of the actual values, and SD denotes the standard deviation.

### 2.7. Software and Configurations

The visualization and hyperspectral image correction were operated in ENVI 5.3. Preprocessing, and the extraction of key wavelength and spatial information and model building were conducted using Python 3.8 on the PyCharm platform. Specifically, when carrying out wavelength selection, the population size and maximum iterations in VSPSO were set to 30 and 200. In model building, the SVR and PLSR models were established with the “scikit-learn” tool (version 0.24.1). The training of LightGBM was implemented with the “lightgbm” tool (version 3.3.5), the grid search for model configuration determination employed “GridSearchCV” in “scikit-learn,” and the determined LightGBM parameters are presented in Table 1. All experimental results were obtained from the average values of ten independent operations.

## 3. Results and Discussion

### 3.1. Statistical Information of Reference Values of TF and TCA Contents

Table 2 presents the statistical information of the reference values of the contents of total flavonoids (TFs) and chlorogenic acids (TCAs) in chrysanthemum tea. The average contents of TFs and TCAs were 126.77 mg/g and 19.57 mg/g. The whole sample set comprised a calibration set (140/200) and a prediction set (60/200). The calibration samples were collected from the blooming period in 2022 and used to conduct the key wavelength selection and model establishment. The external prediction set constituted the chrysanthemums harvested in 2023 and was used to verify the model performance. The corresponding statistical information of both components demonstrated no significant deviation the between the calibration and prediction sets, presenting a relative robustness for subsequent chemometric analysis [47].

### 3.2. Spectral Analysis

**Figure 4:**
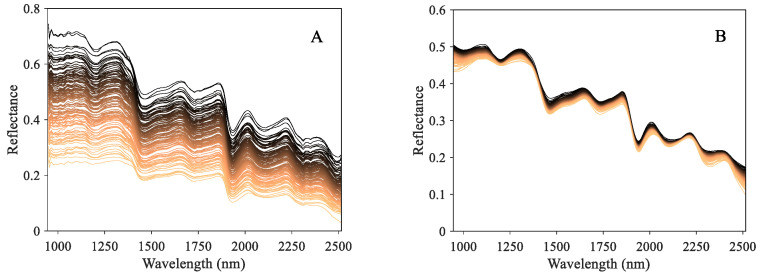
Spectral data of chrysanthemum samples. (**A**) The raw spectral data. (**B**) The spectral data preprocessed by MSC + SGCS.

Figure 4A depicts the raw hyperspectral curves of samples with different bioactive component contents. Among these spectral curves, despite the difference in reflectance strength, they presented the similar absorption peak locations. These distinctive absorption peaks are related to the vibration and stretching modes of functional groups or chemical bonds of phytochemicals [48], thereby indicating consistent interior constituents. Existing studies have revealed that the measurement of characteristic wavelength is suitable for quantitative analysis for specific components [49]. As shown in Figure 4A, the absorption peaks were mainly distributed at 1200, 1460, 1730, and 1930 nm. Specifically, the 1200, 1460, and 1930 nm may be associated with the C-H, O-H, and C=O organic groups [50,51], which are the main components of ketones, carboxylic acids, and their derivatives. The wavelength at 1730 nm is related to the C-H stretching first overtone and –CH_2_ [48], which mainly consists of amino acids and fatty acids. However, despite containing large amounts of information, it is still difficult to accurately and rapidly carry out quantitative analysis using raw spectra directly due to the interference of irrelevant noise and redundant bands [52]. Consequently, preprocessing and key feature extraction are essential for eliminating the noise and heightening the underlying correlation between spectral features and object information.

### 3.3. Analysis of the Preprocessing Methods

Figure 4B depicts the preprocessed spectral curves from the collaboration of MSC and SGCS. Compared with the raw spectra in Figure 4A, it tended to be more concentrated and smoother, presenting an obvious distribution of spectral absorption peaks. It shows that the joint preprocessing approach could take advantage of both MSC and SGCS to decrease the noise interference and enhance the spectral characteristic effectively.

Table 3 presents the performance of the PLSR model for estimating total flavonoid and chlorogenic acid contents using different preprocessing methods. Based on the results, the prediction results of both components using raw spectral data reached R^2^ = 0.8454 and 0.7977, respectively, demonstrating the feasibility of quantitative analysis for chrysanthemum tea with the spectral technique. However, despite the reasonable accuracy, the prediction performance was still expected to be able to be improved further. With the involvement of three preprocessing methods, the PLSR model achieved a superior performance for TF and TCA estimation, and the R^2^ score increased by 0.0106–0.0431 and 0.0262–0.0787 compared to raw spectra. This indicates that the purified data from preprocessing were more effective at enhancing the model performance and alleviating the irrelevant noise interference contained in the raw spectra. Finally, among three used preprocessing methods, the collaboration of Savitzky–Golay convolution smoothing (SGCS) and multiplicative scatter correction (MSC) outperformed the independent employment of each, and achieved the optimal accuracy of R^2^ = 0.8885 and 0.8674. This can be explained by the fact that multiple preprocessing approaches can eliminate the noise interference from various sources. Specifically, SGCS mainly focuses on improving the smoothing characteristic of spectrum curves, thereby decreasing the glitch noise interference, while MSC can alleviate the multiplicative scatter impacts caused by an uneven surface. A similar phenomenon has also been found in [53] (the soluble solid content measurement in mandarin). Consequently, this study employed joint MSC and SGCS as the preprocessing method for subsequent analysis.

### 3.4. Regression Performance of Conventional Models

In this section, several conventional models for bioactive constituent content estimation in chrysanthemum tea were established, including support vector regression (SVR), partial least squares regression (PLSR), and one-dimensional convolutional neural network (1DCNN). Moreover, two prevailing wavelength selection methods were also employed to search for the key wavelengths contributing to quantitative analysis, consisting of the successive projections algorithm (SPA) [54] and uninformative variable elimination (UVE) [55]. Table 4 presents the results of different models and their combination with wavelength selection methods.

From the perspective of models without wavelength selection, SVR performed relatively worse compared with PLSR and 1DCNN in the prediction of both components with R^2^ = 0.8496 and 0.8329. This can be attributed to the simple model structure and mechanism of SVR, which is susceptible to the high-dimensional features and the sample size. In comparison, the 1DCNN model achieved the optimal regression performance. Specifically, the R^2^ values of total flavonoids and chlorogenic acids reached 0.9233 and 0.8956, increasing by 0.0737 and 0.0627 compared with SVR, and the corresponding RMSE values decreased by 1.2499 and 0.0452. A possible reason is the outstanding one-dimensional variable convolution mechanism in 1DCNN, which can adaptively extract the prominent features and exploit the variable relevance despite the requirement of a larger computational consumption [20].

Moreover, regarding the results using wavelength selection, the model performance with UVE was superior to that with SPA and approximated the full spectrum. Specifically, for total flavonoid content estimation, the R^2^ values of UVE-SVR and UVE-PLSR achieved 0.8398 and 0.8567, increasing by 0.0261 and 0.0075 compared with SPA-based models. Similarly, UVE-PLSR achieved a better accuracy for the prediction of total chlorogenic acids content (R^2^ = 0.8581, RMSE = 0.1991, MAE = 0.1696, RPD = 2.6771), outperforming other models using SPA. This can be explained as follows: UVE retains relatively more spectral features compared to SPA. Although SPA can reduce the feature dimension to a lower level, a certain amount of contributing information might be removed simultaneously [56]. Moreover, models using full-spectrum data contain all of the spectral information, thereby achieving superior regression performance.

Overall, the results demonstrate the effectiveness for the estimation of total flavonoids and chlorogenic acids with conventional methods and spectral information. However, there are still several limitations. For instance, the traditional wavelength selection evaluates the variable importance via the absolute values of the regression coefficients while neglecting the combinatorial performance of feature combinations. Furthermore, another advantage of the HSI technique is the contained spatial image information, which might also be conducive to enhancing the quantitative analysis performance [26]. These findings suggest the potential of further improvement with effective feature extraction and model building.

### 3.5. Regression Performance of LightGBM Model with Fused Features

#### 3.5.1. Analysis of the Coefficient of Regularization Item

This study developed a regularization item-based wavelength selection approach (VSPSO) to take advantage of the combinatorial optimization of PSO and remove redundant wavelengths. To investigate the impact of the regularization item coefficient *λ*, we conduct the wavelength selection with *λ* ranging from 0 to 1.

**Figure 5:**
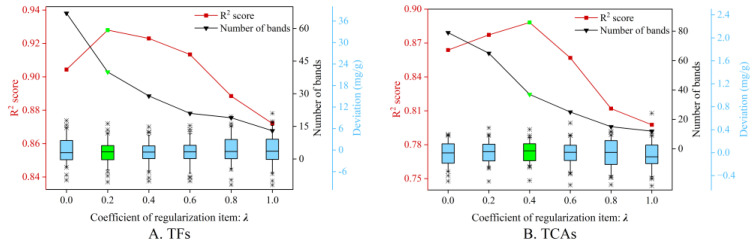
Model prediction results for TFs (**A**) and TCAs (**B**) with different regularization coefficients *λ*. The line marked in red denotes the R^2^ values, and the black one is the number of selected spectral bands. The boxplots are the corresponding prediction deviation when employing different *λ*. The results marked in green denote the obtained optimal performance.

Figure 5 depicts the curves of the R^2^ values and the number of retained bands when employing different *λ* in predicting both components. Specifically, *λ* = 0 corresponds to the case of standard PSO without a regularization item, and achieved R^2^ = 0.9044 and 0.8638 with the most spectral features (67 and 79). This indicates that the standard PSO might be inadequate in reducing the redundant bands despite the decent combinatorial optimization capacity. With *λ* increasing, the number of retained bands started to decline. On the contrary, the R^2^ score tended to rise, and reached the maximum when *λ* = 0.2 and 0.4, respectively, and the number of selected wavelengths was 40 and 37. This phenomenon reveals that the variable similarity-based regularization item is effective at removing unnecessary wavelengths while enhancing regression performance. As *λ* kept rising, both R^2^ and the band quantity were decreasing. It can be explained that the VSPSO with a larger *λ* focuses more on removing the redundant features, while ignoring others to reduce the prediction error. Therefore, this study heuristically set *λ* = 0.2 and 0.4 for the prediction of both components in the subsequent experiments.

#### 3.5.2. Ablation Experiments

This study carried out an ablation experiment to reveal the effects of each module. The results of five modules and their different combinations are presented in Table 5.

Case 1 represents the model performance of LightGBM using raw spectral data. Compared to the model using joint preprocessing by MSC and SGCS (Case 2), the preprocessed data achieved superior accuracy for both components. This phenomenon is consistent with the results in Section 3.3 using the PLSR model, further indicating the necessity of the preprocessing operation to decrease the noise interference before model establishment.

**Figure 6:**
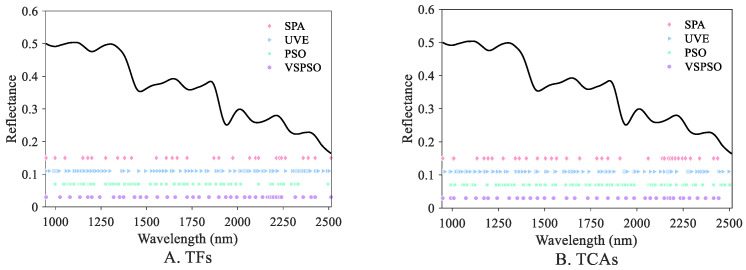
Results of different wavelength selection methods for TF (**A**) and TCA (**B**) prediction.

In comparison with Case 2, Case 3 additionally employed the PSO wavelength selection. Figure 6 depicts the results of key wavelength selection using different methods. Based on the results, despite the decent regression performance of standard PSO, it might still have been insufficient for dimension reduction and removing redundant bands. This can be attributed to the fact that standard PSO typically pays more attention to searching for the appropriate band combination, contributing to decreased prediction error. In comparison, Case 4 introduced the VS regularization term and achieved a better model performance, with R^2^ = 0.9280 and 0.8882, approximating to Case 2, and the number of selected wavelengths was 40 and 37. A possible reason is that VSPSO considers both combinatorial optimization performance of spectral wavelengths and the elimination of collinear features, thereby outperforming Case 3.

Moreover, Case 5 represents the model performance using spatial image features of texture and color information, and achieved the worst regression performance compared with spectra-based models. This is possibly because the spatial information describes the exterior morphological characteristics [39]. Despite the potential contribution to quantitative analysis, it is typically employed as supplementary information for spectral features. Finally, Case 6–8 present the impacts of texture and color information on model performance as the complementary information of the spectral features. Based on the results, the model performance achieved optimal accuracy, with R^2^ = 0.9541 and 0.9137, after the involvement of texture and color information simultaneously. Interestingly, for the independent impacts of texture and color features, the color information was superior to texture information (Case 6 > Case 7). A potential reason is the inherent correlation between the color factors and the concentration of bioactive components. Furthermore, the extracted texture information might have been insufficient to account for the content difference, which is worthy of further study [57].

#### 3.5.3. Model Results and Reliability Analysis

Table 6 presents the model performance of LightGBM and conventional models using the fused features of key wavelengths and spatial image features. First, in comparison with the results presented in Table 4, the models with fused features outperformed the traditional methods using only spectral information. This phenomenon further reveals that the spatial features contained the useful information contributing to the quantitative analysis for internal components. Second, among the employed models, LightGBM achieved the optimal regression performance on the estimation for both bioactive components in chrysanthemum tea. Specifically, for TF prediction, the model accuracy reached R^2^ = 0.9541, RMSE = 2.4150, MAE = 2.0353, and RPD = 4.7095, and the R^2^ value increased by 0.0182–0.0767 compared to other models. Meanwhile, the model performance for measuring TCAs achieved R^2^ = 0.9137, RMSE = 0.1553, MAE = 0.1319, and RPD = 3.4326, with an improvement of 0.0094–0.0582 in the R^2^ value.

Overall, the ensemble learning-based LightGBM exhibited a superior nonlinear fitting capacity compared to SVR, PLSR, and 1DCNN. This can be explained by the following reasoning. Despite the promising potential and more complex structure of 1DCNN, it contains many network parameters, which typically need a large quantity of samples for model training and have a corresponding computational cost. In comparison, LightGBM utilizes the boosting-based strategy to fit the prediction residual error, and develops the GOSS and EFB operations to accelerate the model training [33]. Consequently, with relatively sparser structure parameters, LightGBM presented an outstanding generalization ability.

**Figure 7:**
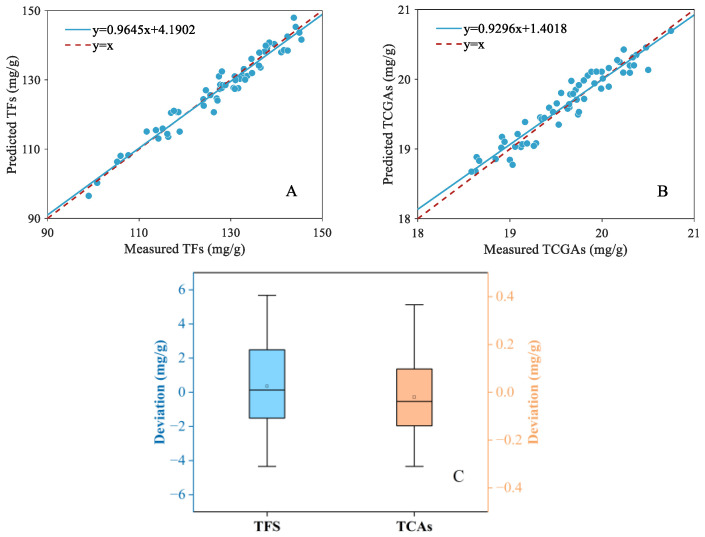
Scatter plots of the predicted and measured values for TF (**A**) and TCA (**B**) content prediction. The boxplot of prediction deviation of TFs and TCAs (**C**).

Figure 7A,B depict the scatter distribution results of LightGBM for estimation of the contents of TFs and TCAs using fused features. Based on the results, the scatter distribution presented a satisfactory regression relationship between the predicted values and the reference with the points close to the 45° line. Figure 7C illustrates the boxplot of the deviation in reference and predicted values. It can be observed that the prediction deviation of TFs was distributed to within ±6 mg/g. The maximum deviation was 5.6760 mg/g, accounting for 3.88% of the average content of the reference values. Similarly, for TCA estimation, the prediction deviation was situated within ±0.4 mg/g. The maximum deviation was 0.3664 mg/g, accounting for 1.74% of the average content of the reference values. Therefore, according to the experimental results, it can be concluded that the LightGBM model using the fused features achieved a satisfactory regression relationship and a lower prediction deviation, presenting a reliable model performance.

## 4. Conclusions

This study developed a novel approach to estimating the bioactive component contents in chrysanthemum tea rapidly and nondestructively by combining HSI and chemometrics. To alleviate the interference of collinear spectral features, a variable similarity regularization item was introduced to particle warm optimization (VSPSO) to remove the redundant features and enhance the combinatorial performance of the selected bands. The experimental results showed that the proposed SPSO achieved superior accuracy for TF and TCA prediction, and the R^2^ score reached 0.9280 and 0.8882 with the LightGBM model. Moreover, to exploit the underlying correlation between the components and the external morphological information, this study also extracted the color and GLCM-based texture features. After the spectral–spatial feature fusion, this study achieved the optimal regression performance for TFs and TCAs, with R^2^ = 0.9541 and 0.9137, respectively, increasing by 0.0308–0.1404 and by 0.0181–0.1066 compared with classical methods and models. Finally, according to the results of the regression relationship and reliable analysis, this study proved the higher performance and reliability for the measurement of bioactive component contents in chrysanthemum tea, which could also facilitate the development of rapid and nondestructive detection in HSI-related fields of agriculture and food industry.

Despite the promising model performance, there might be still limitations discovered in this study. First, the EFB approach enables LightGBM to enhance the model efficiency by bundling mutually exclusive features to the lower-dimensional space. However, it is inevitable that the original feature representation deteriorates. Therefore, the interpretability research is also worthy of studying for understanding the degree of contribution of the sample features explicitly. Moreover, in practical applications, there is also difficulty in data collection and label information acquisition, especially for expensive foods. This might lead to inadequate model training and deteriorate the generalization ability. Considering this limitation, the data generation and enhancement are a promising solution, and will be continued in our future work.

## Figures and Tables

**Table 1:** Grid search space and optimal parameters of the LightGBM model.

Parameters	Search Space	Optimal Parameters	
		TFs	TCAs
num_boost_round	[1, 3000]	412	121
max_depth	[−1, 10]	3	3
learning_rate	[0.01, 0.3]	0.08	0.19
lambda_l1	[0, 1]	0.39	0.47
lambda_l2	[0, 1]	0.07	0
metric	mse		
boosting_type	gbdt		
early_stopping_rounds	100		

**Table 2:** Statistical description of bioactive components in chrysanthemum tea.

Components	Data Sets	Samples	Content Indicators (mg/g)			
			Max	Min	Mean	Std
TFs	Calibration	140	146.20	98.24	125.71	10.39
	Prediction	60	145.45	98.98	127.92	11.37
	Total	200	146.20	98.24	126.77	9.97
TCAs	Calibration	140	21.10	18.45	19.56	0.52
	Prediction	60	20.75	18.58	19.62	0.53
	Total	200	21.10	18.45	19.57	0.52

**Table 3:** Model prediction results of total flavonoids and chlorogenic acids using PLSR with different preprocessing methods.

Bioactive Components	Preprocessing	Evaluation Metrics			
		R^2^	RMSE	MAE	RPD
TFs	Raw	0.8454	4.4339	3.6182	2.5651
	MSC	0.8721	4.0340	3.2663	2.8194
	SGCS	0.8560	4.2797	3.3018	2.6576
	MSC + SGCS	0.8885	3.7658	3.0546	3.0202
TCAs	Raw	0.7977	0.2377	0.1952	2.2419
	MSC	0.8569	0.1999	0.1610	2.6655
	SGCS	0.8239	0.2218	0.1830	2.4031
	MSC + SGCS	0.8674	0.1924	0.1576	2.7695

**Table 4:** Model prediction results of total flavonoids and chlorogenic acids using conventional models and different wavelength selection methods.

Bioactive Components	Models	Evaluation Metrics			
		R^2^	RMSE	MAE	RPD
TFs	SVR	0.8496	4.3734	3.3320	2.6006
	SPA(29)-SVR	0.8137	4.8679	3.7432	2.3364
	UVE(96)-SVR	0.8398	4.5136	3.5128	2.5198
	PLSR	0.8885	3.7658	3.0546	3.0202
	SPA(29)-PLSR	0.8492	4.3793	3.5278	2.5971
	UVE(96)-PLSR	0.8567	4.2694	3.4015	2.6640
	1DCNN	0.9233	3.1235	2.7053	3.6413
TCAs	SVR	0.8329	0.2160	0.1783	2.4670
	SPA(38)-SVR	0.8071	0.2321	0.1929	2.2959
	UVE(89)-SVR	0.8152	0.2272	0.1843	2.3456
	PLSR	0.8674	0.1924	0.1576	2.7695
	SPA(35)-PLSR	0.8382	0.2126	0.1790	2.5068
	UVE(89)-PLSR	0.8581	0.1991	0.1696	2.6771
	1DCNN	0.8956	0.1708	0.1464	3.1209

**Table 5:** LightGBM model results of the ablation experiments.

Case	Modules ^1^					Total Flavonoids				Total Chlorogenic Acids			
	Pre	PSO	VS	Tex	Col	R^2^	RMSE	MAE	RPD	R^2^	RMSE	MAE	RPD
1	×	×	×	×	×	0.8721	4.0340	3.2663	2.8194	0.8260	0.2204	0.1866	2.4177
2	√	×	×	×	×	0.9376	2.8173	2.3618	4.0370	0.9090	0.1594	0.1339	3.3435
3	√	√	×	×	×	0.9044	3.4879	2.8294	3.2608	0.8638	0.1950	0.1637	2.7328
4	√	√	√	×	×	0.9280	3.0263	2.6422	3.7582	0.8882	0.1767	0.1516	3.0161
5	×	×	×	√	√	0.7276	5.8860	4.7830	1.9323	0.6938	0.2925	0.2317	1.8223
6	√	√	√	√	×	0.9433	2.6891	2.2955	4.2369	0.9105	0.1581	0.1329	3.3710
7	√	√	√	×	√	0.9315	2.9526	2.5104	3.8521	0.8948	0.1714	0.1418	3.1093
8	√	√	√	√	√	0.9541	2.4150	2.0353	4.7095	0.9137	0.1553	0.1319	3.4326

^1^ Abbreviations of modules. Pre = preprocessing by MSC + SGCS; PSO = standard PSO wavelength selection; VS = variable similarity-based regularization term; Tex and Col = spatial image features of texture and color information.

**Table 6:** Model prediction results of total flavonoids and chlorogenic acids using different models and fused features.

Bioactive Components	Models	Evaluation Metrics			
		R^2^	RMSE	MAE	RPD
TFs	SVR	0.8774	3.9490	3.1268	2.8801
	PLSR	0.9026	3.5198	2.8071	3.2312
	1DCNN	0.9359	2.8855	2.4218	3.9831
	LightGBM	0.9541	2.4150	2.0353	4.7095
TCAs	SVR	0.8555	0.2009	0.1685	2.6529
	PLSR	0.8740	0.1876	0.1560	2.8409
	1DCNN	0.9043	0.1634	0.1353	3.2621
	LightGBM	0.9137	0.1553	0.1319	3.4326

## Data Availability

The original contributions presented in this study are included in the article. Further inquiries can be directed to the corresponding author.
